# Serving with Pharmacy Students: Reflections from a Medical Mission Team Leader and Preceptor

**DOI:** 10.3390/pharmacy4040033

**Published:** 2016-10-24

**Authors:** Dana A. Brown

**Affiliations:** Pharmacy Practice and Administration, Lloyd L. Gregory School of Pharmacy, Palm Beach Atlantic University, West Palm Beach, FL 33401, USA; dana_brown@pba.edu

**Keywords:** pharmacy, medical missions, pharmacy students

## Abstract

The medical mission field is an innovative setting for training and evaluating health care professional students. The motivating factor of serving indigent populations as a means of a humanitarian, or oftentimes a spiritual act, makes medical missions an attractive option for student participation. At the Gregory School of Pharmacy, medical mission teams are an integral part of the pharmacy program, including the opportunity for students to earn elective credit during their fourth year. This commentary provides five key elements to consider when serving with, training and evaluating pharmacy students from the perspective of a team leader and preceptor.

## 1. Introduction

Medical missions are an established means of providing health care in desolate areas of the world [1]. These experiences can be prime opportunities to train pharmacy students given the vast array of drug-related challenges that demand a solution. The motivating factor of serving indigent populations as a means of a humanitarian, or oftentimes a spiritual act, makes medical missions an attractive option for student participation. How does a faculty member or pharmacist facilitate this opportunity for student growth as well as their own personal and professional growth? To answer this question, I will share with you some of my experiences on the mission field with students, serving as both the team leader and preceptor.

At the Gregory School of Pharmacy at Palm Beach Atlantic University (PBA), medical missions is an integral part of the Doctor of Pharmacy program. Although not required of students, both international and domestic medical mission experiences are encouraged and have become inculcated into the culture of the School. These experiences compliment the model of *servant leadership* that is integrated throughout the curriculum. In addition, they are fully aligned with our mission, which states: “We develop servant-leaders who are fully committed to raising the standards of practice within the profession of pharmacy by following Christ’s example of serving, teaching, and healing those in need.” Fourth year students may take an elective advanced pharmacy practice experience (APPE) in medical missions to receive academic credit. As part of this elective APPE, they attend one trip at the start of their fourth year where they involved with various projects and experiences including, but not limited to, case presentations, formulary management, dispensing and compounding, and training in skills such as blood pressure and blood glucose assessment. Preceptors utilize the fourth year pharmacy students as a means of leadership and training within the team. The Global Pharmacy Education Special Interest Group as part of the American Association of Colleges of Pharmacy released current practices for pharmacists and students when completing APPEs globally [2]. The faculty at PBA use these to guide the planning and execution of the mission APPE.

The first missions experience with the School occurred in 2003. A total of 44 as of the summer of 2016 have occurred, reaching 14 locations across the United States and the world (See Table 1). Data collection of experiences began in 2008, and from this we have determined that a total of 501 students have participated over this time span with efforts reaching 12,773 patients and 32,033 prescriptions and over-the-counter products dispensed. While most of these experiences are led by faculty, some alumni have been involved as leaders as well as team members. We have also worked with the PBA’s nursing program. We are expanding on this collaboration along with other professions as a means of building our interprofessional education. In addition, students from other schools of pharmacies have also participated on our medical missions teams.

Because of the expansion of the School’s missions program, policies and procedures have been developed to specifically articulate the expectations of the students, faculty and alumni leading and participating in these experiences. Although this is not a routine part of team determination, interviews and essays have been used on a few occasions. An application and fee is part of the process. Although we plan our missions experiences internally, we partner, in almost all cases, with a mission organization to manage the logistics of the team such as flights, lodging, food and transportation. Because our teams are composed of almost all pharmacists or pharmacy students, it is imperative that our contacts in each country are able to arrange for local physicians, nurses and/or other health care practitioners to work alongside the team in order to effectively carry out medical clinics. Most of the settings our teams work within are ambulatory care in nature. However, a few teams have served in institutional settings. Another model that pharmacists may be involved with on the mission field is working with medical mission organizations directly to fill a need. For example, organizations such as Global Health Outreach arrange numerous medical mission trips per year, and they seek volunteers from a variety of medical professions to participate on these teams [3]. This model seeks to determine the right balance of health care practitioners in order to provide the most holistic care in each country as opposed to relying solely on other health care professionals in each country. If this model is chosen, it is important to predetermine if pharmacy students are able to join the team under the supervision of a licensed health care practitioner on the team. Other team models to consider include establishment of a home-grown team to work with key contacts in the destination country or partnership with a governmental agency to serve within existing agencies, amongst others.

## 2. Key Elements for Serving with Students on the Mission Field

The Gregory School of Pharmacy offers three to five medical mission experiences each year. During my tenure at PBA, I have served on seven international medical mission teams, six of which I was a team leader and preceptor. As part of this experience, I have identified five key elements to consider when serving with, training and evaluating pharmacy students within a predominantly ambulatory care setting.

### 2.1. Start Planning Early

If there is one thing I have learned about being a team leader, it is the importance of beginning to plan the experience as soon as you are aware that you will be going. There is more involved in planning and executing an experience than most realize on the front end. Once an experience has been established, whether this experience is occurring internally through the organization or remotely with a mission organization, it is important to establish deadlines for applications as early as possible. Some applications require references, a notary seal, essays, amongst other things that require time and effort. Fundraising by the team can be critical if support funding is not being sought. Therefore, planning is important to maximize the funds to be raised. Clear expectations as to how the money will be utilized should be established up front to avoid any miscommunication about the allocation of those funds. In addition, it is important to plan for students who may withdraw from going on the trip despite payments that have been made. Whether this is due to personal reasons, illness, or other obligations, determining what happens when a student withdraws from an experience, particularly with regards to their funding, is critical. At PBA, we do not reimburse funds once airline tickets have been purchased. Instead, we seek to find a replacement for the student’s slot and have them work out payment issues between each other.

Also, as pharmacists, we should play an integral role in formulary development and management. As the manager of the formulary, the pharmacist should be responsible for the distribution of the formulary to all prescribers on the team as well as educating the team on any medications that may be unfamiliar. Several organizations such as Christian Pharmacists Fellowship International have example formularies [4]. From a personal perspective, first time experiences are often a “trial and error” method for determining which medications are most utilized. Communication with local doctors may help drive some formulary decisions. Recognizing how much of the budget is devoted to medications is critical so that the appropriate allocation of funds can be used for this purpose. Numerous organizations such as Blessings International and Kingsway Charities exist which provide medications at a reduced cost for medical missions efforts [4,5,6]. MAP International (Brunswick, GA, USA) offers pre-packed medication boxes which contain the “essentials” needed for medical mission experiences [7]. Upon returning from the field, it is important to quickly reflect on the medication formulary to help evolve it for the following year if there are plans to return to the same location.

### 2.2. Meet Routinely, If Possible

If the team members are geographically able, routine meetings should ideally occur every one to two weeks based on past experiences. These allow for the timely dissemination of information and deadlines as well as answering any questions that may arise. Meetings have also been useful for training pharmacy students on the various patient care activities they encounter. For my most recent experience, I required the fourth year pharmacy students to train/refresh the second and third year students on blood glucose and blood pressure techniques. They were also involved with explaining the logistics of our clinic and how to perform a patient assessment based on the forms our team was using.

Perhaps most importantly, meeting routinely begins to strengthen team dynamics. Team building activities are recommended as a means to augment team dynamics. If the team members are unable to gather in a physical location for a meeting, communicating through technology is another means for hosting meetings. Establishing social media connections through team pages and groups also helps to promote communication.

### 2.3. Establish Clear Expectations and Define Roles

Well-articulated expectations in regards to attendance at team meetings, participation on the trip, assignments (for students receiving academic credit), financial information, amongst others are critical, especially when working with students. Holding students accountable in a consistent manner to meeting these expectations is equally as important. When students sign up for a trip and make a commitment to participate, they should each be able to clearly articulate what is expected of them by participating on the medical mission team. The functions of a team require just that—a team of individuals. When members do not follow through with their commitment, the infrastructure of the team is jeopardized which can create moments of disunity on the team.

When working on interprofessional teams, defining roles up front is important to ensure smooth logistics on clinic days. This might be even more important if there are overlapping roles, such as blood pressure assessments. If multiple people are qualified and trained to do the same activity or perform the same task, which is almost always the situation in teams I lead, designing a schedule to rotate team members has been beneficial to ensure everyone gets an equal opportunity to participate as well as experience the various aspects of the clinic. For example, in our teams, we have students rotating through triage, the doctor visit, pharmacy, patient counseling, and children’s ministry. This gives everyone an idea of what happens with each piece of the clinic day and helps to provide important feedback for improving services. The areas of service may differ depending on the type of setting the team is working within. For example, triaging may be staged differently for an ambulatory care team as opposed to a surgical team.

### 2.4. Put the Students in the Trenches

Pharmacy students are not licensed to provide medical care; therefore, they are under the supervision of the pharmacist(s) they are working with. As such, it is important for the pharmacist(s) to “roll up their sleeves” and model the activities expected of the students. While it is certainly easier at times just to complete the activity as the pharmacist, resist the temptation to do it yourself and let the students experience it. The application of what was learned in the classroom is a critical component in the training of pharmacy students. Give the students opportunities and projects that will result in critical thinking such as performing calculations, compounding a suspension, or determining the most appropriate medication for a given patient. As part of pre-experience training, we discuss ethical situations that students might be faced with, such as incestual relations leading to pregnancy in a young girl and when to seek guidance from the preceptor. Working closely with students in challenging situations is a dynamic way for students to see the application of ethical decision making. The mission field is a great place for this practice and application to take place as they will see more patients in one setting over the course of one day than they likely would in a traditional pharmacy retail or ambulatory care setting in the Western world.

### 2.5. Encourage and Praise the Students

The mission field is where some of my deepest and most lasting relationships have been developed, with other health care practitioners and students. Serving an indigent population in an area that is most likely out of a comfort zone brings unity in many cases. While the experience might be overwhelming at first, students generally acclimate to the environment rather quickly, often not by their own choice! As I am working alongside students, I have discovered that praise and encouragement of the tasks and activities they are completing pays dividends. A simple “great job of counseling that patient” can help boost confidence in the most insecure student. It may also increase their productivity during the team’s activities on the trips. Upon return from the experience, I find that students on teams I have led often gravitate to me for letters of recommendation, seek personal advice and remain in touch, even after graduation.

When students are not performing to expectations or not conducting themselves in a professional manner, it is equally as important to provide open, honest constructive feedback. Once the deficiencies have been identified and corrected, praise and encouragement can be shared upon meeting these expectations.

## 3. Conclusions

The medical mission field is a ripe location to train pharmacy students, while helping a group of people in tremendous need. Students are often given ample opportunities on the mission field to explore classroom knowledge with “real patients”. Amply preparing for the mission field can help team leaders effectively lead, precept, and inspire their students.

## Figures and Tables

**Table 1:** Frequency and location of medical mission trips at the Gregory School of Pharmacy from 2003 to summer 2016.

Frequency	Location
1	Alaska
3	Amazon River
2	Belize
6	Belle Glade, Florida
3	Bolivia
9	Costa Rica
6	Dominican Republic
1	Ecuador
2	Guatemala
1	Haiti
4	Honduras
1	Taiwan
4	Uganda
1	Zambia
